# Effect of Moxidectin Treatment at Peripartum on Gastrointestinal Parasite Infections in Ewes Raised under Tropical Andes High Altitude Conditions

**DOI:** 10.1155/2015/932080

**Published:** 2015-05-11

**Authors:** J. J. Vargas-Duarte, H. Lozano-Márquez, H. A. Grajales-Lombana, C. Manrique-Perdomo, D. A. Martínez-Bello, C. Saegerman, M. Raes, N. Kirschvink

**Affiliations:** ^1^Genetic Institute, National University of Colombia, Carrera 30 No. 45-03, Edificio 426, Bogotá D.C., Colombia; ^2^Unit of Integrated Veterinary Research, Department of Veterinary Medicine, University of Namur, rue de Bruxelles 61, 5000 Namur, Belgium; ^3^Faculty of Veterinary Medicine and Animal Science, National University of Colombia, Carrera 30 No. 45-03, Edificio 481, Bogotá D.C., Colombia; ^4^Faculty of Veterinary Medicine, Cooperative University of Colombia, Calle 30 No. 33-51, Bucaramanga, Colombia; ^5^Research Unit of Epidemiology and Risk Analysis Applied to Veterinary Sciences (UREAR), Fundamental and Applied Research for Animal and Health (FARAH), University of Liège, boulevard de Colonster 20, 4000 Liège, Belgium

## Abstract

This study tested the impact of moxidectin at peripartum on nematode fecal egg count (FEC) and clinical parameters on ewes in the high altitude tropical Andes of Colombia. FEC and clinical evaluations were performed on 9 occasions in 43 naturally infected ewes before and during gestation and after lambing. Moxidectin (Mox, 200 *µ*g kg^−1^) was applied at late pregnancy (*T*_1_, *n* = 15) or 48 hours after parturition (*T*_2_, *n* = 14). 14 untreated ewes served as controls (C). Suckling lambs (*n* = 58) remained untreated and underwent four clinical and parasitological evaluations until 8 weeks after birth. Mox efficacy equaled 99.3% (*T*_1_) and 96.9% (*T*_2_). Highest mean FEC value reflecting periparturient nematode egg rise (PPER) was recorded in C ewes at 4–6 weeks after lambing. Significant FEC reductions were found in *T*_1_ (94.8%) and *T*_2_ (96.7%) ewes (*p* < 0.05). All lambs showed a significant and ewes-group independent increase in FEC before weaning (*p* < 0.05). Clinical parameters (anemia and diarrhea) showed time- and treatment-related differences (*p* < 0.05). Monitoring of FEC and clinical parameters linked to gastrointestinal parasite infections allowed demonstrating that postpartum or preweaning are two critical periods to nematode infection for sheep raised under tropical Andes high altitude conditions. Use of Mox as anthelmintic treatment prevented PPER.

## 1. Introduction

Sheep parasites belong to the main constraints that reduce sustainability of wool, milk, and meat production worldwide [1–3]. To prevent parasite dissemination in small ruminant flocks, assessment of the magnitude of gastrointestinal parasite burdens in different productive categories is required [4, 5]. Indeed, lambs have been described as the most susceptible category to gastrointestinal parasites and it is assumed that adult animals can deal with parasite infection and minimize its pathogenic activity [5, 6]. However, it has been shown that periparturient ewes are highly susceptible to gastrointestinal nematode infections and are the largest contributors to pasture contamination with nematode eggs [7, 8].

In mature ewes, a transient loss of immunity to gastrointestinal nematodes begins around lambing time and continues for several weeks after parturition. This impaired resistance is associated with an increase in strongylid fecal egg count (FEC) commonly referred to as the periparturient nematode egg rise (PPER) [5, 9–14]. PPER has been described mainly in sheep breeds exploited in temperate regions. Indeed, the flocks' seasonal management allows the spring contamination of pasture with nematode eggs shed by lactating ewes and the concomitant infection of lambs with the infective larvae hatched from those eggs [15, 16]. Although well known, the exact cause of PPER remains poorly understood. Increased FEC has been also associated with variations in hormonal profiles at peripartum (prolactin and cortisol levels) and low levels of metabolisable protein intake during late pregnancy and lactation [13, 17–21].

In tropical regions of South America, sheep production is based on extensive grazing with limited management and sanitary practices, which leads to increased mortality rates and reduced productivity due to gastrointestinal nematodes [22–24]. Under these conditions, the importance of a PPER and its epidemiological consequences in small ruminants remain to be established [7, 25–28]. On the one hand, infective larvae may be continuously available in the tropics and reproductive cycles and lambing are not strictly seasonal events as in temperate countries. Indeed, in Colombian flocks, lambing might occur during the entire year, but two main annual periods of births during April–June and November-December at the end of rainy periods are described [29]. On the other hand, physiopathological consequences of PPER in lactating ewes and their offspring are believed to be important in the tropics because the infectious pressure persists at a high level and because nutritional management is often heterogeneous [24, 26, 30, 31]. Consequently, environmental parameters, reproductive cycle features, and feeding management practices are fundamental tools for rational anthelmintic treatment [3, 4, 32]. In order to minimize nematode egg outputs and to regulate pasture contamination with infective third larval stages in tropical sheep production systems, strategic treatment of highly infected animal groups, as periparturient ewes, is recommended [2, 8, 33, 34].

Among the available chemical groups used to control gastrointestinal nematodes in sheep, macrocyclic lactones represent the cornerstone of current anthelmintic drug control [35]. Moxidectin (Mox), a macrocyclic lactone of the chemical family of milbemycins, has been widely used during the last 30 years [36]. However, the intensive use of this broad-spectrum antiparasitic compound could lead to the emergence of resistance in gastrointestinal nematodes [35, 36]. This situation has encouraged the search for treatment strategies to optimize Mox potential, avoiding unnecessary treatments, particularly in geographic areas where Mox use is not a frequent practice and resistance is not yet fully present [36, 37].

The present study hypothesized (1) that PPER occurs in Colombian sheep raised under Andean high altitude tropical conditions during the period of the highest lambing rate (April–June), (2) that Mox is still an effective anthelmintic molecule at tropical Andes, and (3) that Mox strategic treatment of ewes administered either at late pregnancy or early peripartum period would prevent PPER.

## 2. Materials and Methods

The study was conducted at the Center for Sheep Research, Technological Development and Extension of the National University of Colombia, located in Mosquera-Cundinamarca (4°40′57′′N, 74°12′50′′W) at 2510 m above sea level. The mean temperature during the study period was 13.5°C and the monthly average rainfall 86 mm. The protocols employed followed the national guidelines for care and use of animals (Colombian Law 89/1989) and were approved by the Ethical Committee of the Faculty of Veterinary Medicine and Animal Science of National University of Colombia (CB-035-2013).

### 2.1. Animals

This study enrolled 43 healthy wool ewes of four breeds (Colombian creole, Romney Marsh, Hampshire, and Corriedale). Ewes were grazing together in* Lolium perenne* and* Pennisetum clandestinum* pastures and were supplemented with concentrate feed, hay, and mineralized salt according to physiological requirements. Fresh drinking water was available* ad libitum*. Ewes' clinical and parasitological follow-up was performed during one complete reproductive cycle (dry period, mating period, pregnancy period, and postpartum period). Since birth, lambs (*n* = 58) were kept with their dams and underwent the same follow-up.

### 2.2. Experimental Design

**Figure 1:**
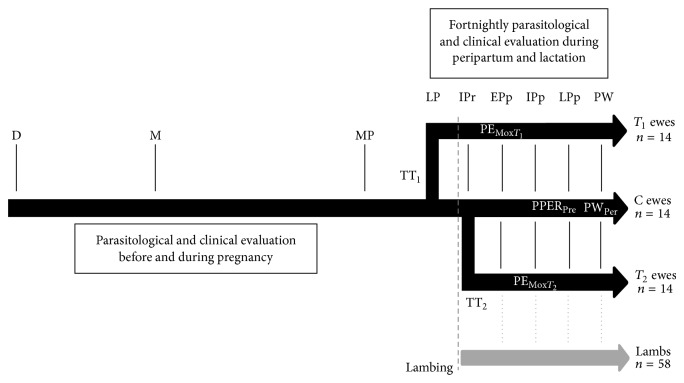
Experimental design to describe periparturient nematode egg rise (PPER) of ewes naturally infected with gastrointestinal nematodes and to measure the efficacy and persistency of peripartum strategic treatment with moxidectin (Mox) either at the onset of late pregnancy (TT_1_) or at the end of immediate peripartum (TT_2_) period under tropical Andes high altitude conditions. Parasitological and clinical evaluations included FEC by McMaster test, anemia detection by FAMACHA© system, diarrhea assessment by Dag scoring, body weight, and body condition assessment. Fecal egg count reduction (FECR) was employed to test posttreatment moxidectin efficacy 15 days after treatment (PE_Mox*T*_1__ and PE_Mox*T*_2__), the prevention of PPER at late postpartum (PPER_Pre_) period, and preweaning persistency (PW_*Per*⁡_). Ewes in control group (C) and offspring of all ewes were untreated against gastrointestinal nematodes. D: dry ewes; M: mating; MP: midpregnancy; LP: late pregnancy; Ipr: immediate postpartum; Epp: early postpartum; Ipp: intermediate postpartum; LPp: late postpartum; and PW: preweaning.

A longitudinal study was conducted during 40 weeks and included nine sampling periods where parasitological and clinical evaluations were performed in relation to ewe's reproductive state (Figure 1). The last anthelmintic treatment was applied more than four weeks before the beginning of the follow-up (fenbendazole; 10 mg·kg^−1^ once daily during three days). Ewes were investigated before breeding, around mating (natural reproduction with a mating period of 34 days), at midpregnancy (~80 days of pregnancy), at late pregnancy (7–29 days before lambing), at immediate peripartum (6 days before until 2 days after lambing), at early postpartum (2–15 days after lambing), at intermediate postpartum (15–30 days after lambing), at late postpartum (30–45 days after lambing), and at preweaning (46–60 days after lambing). Lambs were investigated since birth at the same time points as their dams. At late pregnancy, ewes were ranked on the basis of body weight, body score, and parasite infection burden and allocated into three groups. Ewes of group *T*_1_ (*n* = 15) received a single Mox (Cydectin, Fort Dodge Animal Health) subcutaneous injection of 200 *μ*g kg^−1^ of body weight at late pregnancy (~135 days of gestation). Ewes of group *T*_2_ (*n* = 14) received a single Mox subcutaneous injection of 200 *μ*g kg^−1^ body weight 2 days after lambing. Control ewes (C group, *n* = 14) remained untreated throughout the whole study. Five further fortnightly samplings were performed after lambing.

### 2.3. Clinical and Zootechnical Parameters

The animals were monitored daily during the entire course of the experiment. Development of clinical signs related to parasite infections (depression, ataxia, and/or submandibular edema) was monitored. Body weight and body condition were recorded monthly. Except for dry period, scores of anemia (FAMACHA©) and diarrhea (Dag score) were measured at each sampling period according to Broughan and Wall [38], Di Loria et al. [39], and Macarthur et al. [21] recommendations.

### 2.4. Parasitological Follow-Up

Parasite burden determination was performed by fecal sampling in all ewes at the 9 established periods and in lambs at four occasions. FEC were obtained by the modified McMaster test according to the methodology described by Henriksen and Christensen [40] with a minimum detection level of 50 eggs per gram of feces. In cases of parasite burdens higher than 4000 strongylid eggs per gram and associated clinical signs of nematode infection (FAMACHA© level ≥4 or elevated Dag score), C ewes and/or the untreated lambs were selectively treated and withdrawn. Additionally, lambs whose fecal oocyst count was higher than 4000* Eimeria* oocysts per gram of feces, received an oral toltrazuril anticoccidial treatment (Coccicalf, California Company S.A., 20 mg kg^−1^ of body weight).

### 2.5. Statistical Analysis

To estimate FEC reduction (FECR) induced by treatments, the equations recommended by Dobson et al. [41] were employed to calculate the following.

*(1) Mox Posttreatment Efficacies*. Consider PE_Mox_ = 100 × [1 − (*T*_*i*15_/*T*_*i*0_)×(*C*_0_/*C*_15_)], where *T*_*i*0_ and *T*_*i*15_ are the arithmetic means of pretreatment and 15-day posttreatment FEC of treated groups (*T*_1_ or *T*_2_) and *C*_0_ and *C*_15_ are the arithmetic means of C group at the corresponding sampling times. Mox resistance was suspected when posttreatment FECR was less than 95% and resistance was declared when the upper 95% confidence interval of the percentage reduction was less than 95% [42].

*(2) PPER Prevention Induced by Mox Treatments.* Consider PPER_Pre_ = 100 × [1 − (*T*_*i*LPp_/*C*_LPp_)], where *T*_*i*LPp_ and *C*_LPp_ are late postpartum arithmetic means of FEC in treated (*T*_1_ and *T*_2_) and C groups, respectively. 

*(3) Preweaning Mox Treatments Persistency.* Consider PW_*Per*⁡_ = 100 × [1 − (*T*_*i*PW_/*C*_PW_)], where *T*_*i*PW_ and *C*_PW_ are preweaning arithmetic means of FEC in treated (*T*_1_ and *T*_2_) and C groups, respectively.

Resampling-bootstrap method was employed to provide 95% confidence intervals for anthelmintic efficacies [41]. The package “eggCounts” in R software version 3.1.0 was employed [43].

Statistical analysis of data was conducted employing a two-factor ANOVA with repeated measures on one factor in order to evaluate the effect of both treatment and sampling time on FEC and clinical parameters. If necessary, logarithmic transformation was performed in order to achieve a normal data distribution. Clinical variables as FAMACHA©, Dag score, and body condition were discriminated into categories and presented as median (minimum and maximum values). A ratio change for each clinical and zootechnical variable was calculated by dividing the median values before treatment (dry, mating, midpregnancy, and late pregnancy) over the median after treatment (early, intermediate, late, and preweaning period). Group effect on ratio changes was assessed by one-factor ANOVA and Tukey tests. *p* values less than 0.05 (*p* < 0.05) were considered significant. Statistical analysis was performed using the software SPSS Statistics 21.0.

## 3. Results

**Figure 2:**
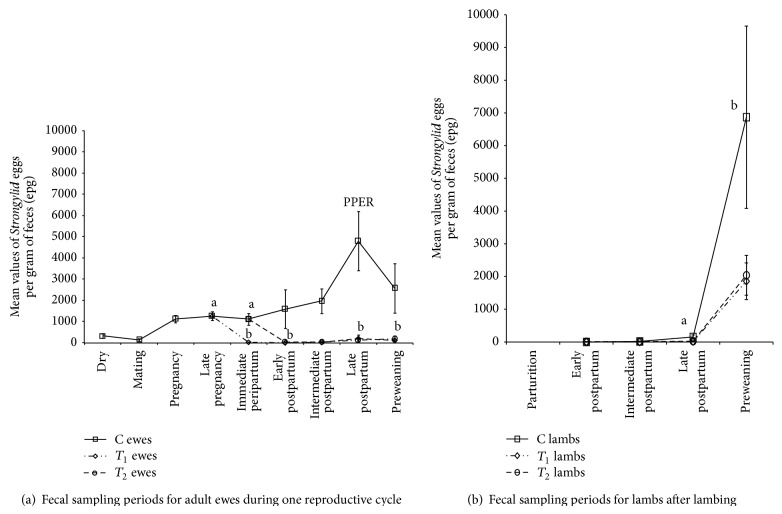
Mean gastrointestinal parasite burdens (± standard error of mean) registered during one complete reproductive cycle in ewes and their offspring. (a) shows the peak in FEC at late pregnancy in C ewes (PPER) and the significant (^b^) and sustained FECR induced by *T*_1_ and *T*_2_ peripartum strategic treatment with moxidectin (two-factor ANOVA, *p* < 0.05) preventing the PPER (PPER_Pre_). (b) shows the significant increase in FEC at preweaning (^b^) in ewes' offspring (two-factor ANOVA, *p* < 0.05). Adult ewes *n* = 43 (14 C, 15 *T*_1_, and 14 *T*_2_). Suckling lambs *n* = 58 lambs (18 born from C ewes, 20 from *T*_1_ ewes, and 20 from *T*_2_ ewes).

An effect of treatment and sampling time on FEC was established (*p* < 0.05). Mean FEC from ewes before and during mating period were significantly lower than those registered during pregnancy and lactation in C ewes. PPER occurred in C ewes at late postpartum period, 4–6 weeks after lambing, and induced a significant increase in FEC (*p* < 0.05) (Figure 2(a)).

Peripartum Mox treatment in *T*_1_ and *T*_2_ ewes significantly reduced FEC 15 days after treatment and efficiently prevented PPER (*p* < 0.05). No differences between *T*_1_ and *T*_2_ mean for FEC after treatment were observed; FEC remained low until preweaning period in both groups.

Significant differences by treatment and sampling time were recorded for clinical parameters (*p* < 0.05). Mox treatment applied to group *T*_1_ prevented increase in FAMACHA values after lambing as observed in C and *T*_2_ groups (Table 2). No major changes were found for body weight and body condition scores.

A significant increase in FEC occurred to lambs at preweaning period (Figure 2(b)), two weeks after FEC peak in control ewes (*p* < 0.05). Although no significant differences among preweaning FEC were observed between lambs born to C, *T*_1_, or *T*_2_ ewes (*p* < 0.05), untreated control ewes offspring showed a sharper increase in FEC (Figure 2(b)) and required treatment. Lambs' clinical parameters did not show significant differences over time or in function of ewes' treatment group (data not shown).

## 4. Discussion

The present study evaluated under field conditions the magnitude of PPER of gastrointestinal nematodes in Colombian wool sheep and tested the efficacy of Mox and its impact on two periparturient treatment schemes in ewes at lambing at the period of the highest birth rate in Andean flocks. PPER and treatment efficiency and persistency were evaluated in terms of FEC as well as in terms of clinical (anemia and diarrhea score) and zootechnical (weight, body condition) performances.

The study was conducted under field conditions with naturally infected ewes. As the animals belonged to the Sheep Research Center of National University in Bogota, they had been selected among a larger group in order to allow an optimal standardization of the study conditions and the investigation of a reduced number of animals (14-15 ewes per group). Local (Creole) or well adapted breeds (Romney Marsh, Hampshire, and Corriedale) were kept on typical Colombian altitude pastures. If keeping all animal groups (*T*_1_, *T*_2_, and C) on the same pasture allowed an optimal standardization of the infectious third larvae pressure, it also diminished the effect of the treatments that were tested. Indeed, in spite of the high efficacy and persistence of Mox strategic treatment, it is likely that FECR observed in ewes would have been even better if separated pastures would have been used after treatment. It might also be assumed that the lack of zootechnical impact (body weight and body condition score) of the treatments was at least partially due to standardization of the infectious pressure. Keeping the animals on separated pastures would have increased group-related differences among ewes and particularly among lambs. However, our study aimed to simulate local breeding conditions, that is, extensive grazing and no separation of treated from untreated animals. Untreated animals favor the “*refugia*” population of parasites by inducing a dilution effect on eggs shed by treated animals, although most treatment practices still rely on systematic treatment of animal groups [44].

The fluctuation in FEC observed throughout ewe's reproductive cycle might have been linked to ewe's productivity stage and the endocrine, immunological, and metabolic changes [6, 13, 16]. Low mean FEC in mature ewes at dry and mating periods could be related to minimum maintenance nutritional requirements and to the active immunological response described against gastrointestinal nematodes in empty adult ewes that limit the establishment of consumed infective larvae and the development and reproduction of parasitic stages [13, 18].

In contrast, nutritional requirements are increased during pregnancy and late pregnancy; hence, it is possible that the increased mean FEC recorded during pregnancy but especially at late pregnancy could be related to higher nutritional demands leading to augmented intake of contaminated herbage with infective larval stages but also to the onset of immunity breakdown towards gastrointestinal nematodes. In this study, the highest percentage of ewes with body condition values ≥3 was recorded at late pregnancy period (88.4%). Macarthur et al. [14] have described that ewe's body condition values ≥3 at late pregnancy improve the productive performance of lactating ewes and suckling lambs and reduce the risk of immune relaxation to gastrointestinal nematodes during peripartum period. This suggests that an adequate nutrient supply by pasturing and supplementation in the flock could reduce the impact on the severity of gastrointestinal infections during lactation. It has been also established that immunological deficiencies against gastrointestinal nematodes start at late pregnancy and they were related to low levels of both circulating eosinophils and total antibodies directed against nematodes 23 days before lambing [45]. The FEC values observed in this study at late pregnancy (Table 1) suggest the need to establish strategic treatment protocols at this critical period, reducing pasture contamination and offspring infection after lambing.

At immediate peripartum period, there was a slight reduction in FEC in control ewes, followed by a significant increase during lactation (Figure 2(a)). On the contrary, ewes of groups *T*_1_ and *T*_2_ displayed a significant and sustained decrease in FEC after periparturient treatment. Although *T*_1_ ewes maintained a slightly lower gastrointestinal nematode burden during all lactation, no differences in terms of FEC or zootechnical parameters were observed between groups *T*_1_ and *T*_2_.

At intermediate and late postpartum period a continuous and drastic increase in mean FEC was observed in control ewes. At this time ewes should increase their catabolism of fat tissue in order to ensure the production of milk to support their lambs [21]. Late postpartum FEC peak reached in control ewes coincides with the systemic and local relaxation of the immunological response against nematodes reported by Beasley et al. [45] six weeks after lambing. It has been described that during these periods the rate of establishment of adult parasites is increased and the ewes lost their ability to suppress nematodes fecundity allowing a further significant increase in FEC [21, 45]. The stress caused by birth process, nursing, and maternal behavior could favor the already depressed systemic immunity and promote the PPER [14]. Indeed, relaxation of immunity against gastrointestinal nematodes was observed in control ewes, in which 35.7% (*n* = 5) had FEC higher than 4000 eggs per gram at late postpartum period and 21.4% (*n* = 3) registered FAMACHA© values ≥4. Only one anemic ewe was recorded in *T*_2_ group.

The significance of PPER observed at late postpartum period in this trial contributes to high larvae pasture contamination especially when susceptible lambs are grazing alongside the ewes, thereby increasing their chances of becoming heavily infected. Our results showed that after the late postpartum peak in C ewes there was a significant increase in lambs' FEC at preweaning period, due to the ingestion of pasture contaminated with nematode infective larvae. An effective acquired immunological response in lambs is only gradually developed during the first year of life [6]. Strategic treatment of ewes seemed to somewhat protect their lambs delaying nematode infection (Figure 2(b)). It is possible that delayed egg excretion in lambs born from treated ewes was due to prolonged Mox secretion by milk suckling. Imperiale et al. [46] showed that residual concentrations of Mox were recovered in milk up to 35 days after treatment (between 17.8 and 183.5 ng mL^−1^ daily). Considering that daily milk intake ranges from 1000 to 2000 mL in lambs, milk secretion of 100 ng mL^−1^ Mox per day would lead to ingestion of 100–200 *μ*g Mox per lamb per day. Although this temporal excretion of Mox could reduce FEC, it also exposes lambs' nematodes to subtherapeutic doses reducing “*refugia”* population of parasites and predisposing to resistance processes [47]. Recently Dever and Kahn [48] have demonstrated that anthelmintics extremely lipophilic as Mox administered at the rate of 1 mg kg^−1^ of body weight to lactating ewes could reduce significantly FEC in suckling offspring and expose lambs to subtherapeutic doses of the drug, a risk factor for the development of anthelmintic resistance. Our study employed only 200 mg mL^−1^ and due to differences in treatment period application (late pregnancy or immediate peripartum), *T*_1_ offspring received lower Mox by milk than *T*_2_. In order to reduce the risk of milk Mox residues, late pregnancy treatment 35 days before lambing could be recommended.

As mentioned earlier, all animals enrolled in this study were kept together in order to standardize infectious larvae pressure and to favor “*refugia*” gastrointestinal nematode population. Despite the high estimated PE_Mox_ and PW_*Per*⁡_, it would however be interesting to assess the protective effect of ewes' peripartum treatment in animals housed on separate pastures in order to evaluate the impact of treatment on FEC and clinical parameters in ewes and lambs whose infectious larvae pressure would be lower than in the present study where untreated control ewes continued to contaminate the environment of treated ewes and their untreated offspring.

## 5. Conclusions

This study describes the FEC changes throughout ewes' reproductive stages and confirmed that PPER exists under tropical Andes high altitude conditions in Colombia. Mox treatment applied prior to or shortly after lambing efficiently prevented PPER and reduced changes of FAMACHA and Dag scores over time. FEC increase occurring in suckling lambs at preweaning period tended to be delayed by treating ewes at peripartum period. Although larger animal groups are needed to characterize the impact of PPER on zootechnical parameters, this study suggests that ewes raised under tropical Andes high altitude conditions are prone to undergo changes of pathophysiological indicators in response to increased gastrointestinal nematode burdens that could be useful in targeted treatment strategies.

## Figures and Tables

**Table 1:** FECR induced by moxidectin strategic treatment of periparturient ewes during the same reproductive cycle.

Time of treatment	Evaluated period	Mean FEC ± SEM		Efficacy of treatment	
		Untreated control ewes *n* = 11^∗^	Treated ewes *n*_*T*_1__ = 15 and *n*_*T*_2__ = 14	% FECR	95% CI
Before lambing (*T*_1_)	Before treatment	1705 ± 537	1060 ± 314	—	—
	15 days after treatment (PE_Mox_)^∗∗^	745 ± 351	3 ± 3	99.3	97–100
	Late postpartum (PPER_Pre_)^∗∗^	4168 ± 1417	217 ± 172	94.8	66.2–99.6
	Preweaning (PW_Per_)^∗∗^	2582 ± 1205	123 ± 59	95.2	79.4–98.9

48 h after lambing (*T*_2_)	Before treatment	1277 ± 491	1043 ± 358	—	—
	15 days after treatment (PE_Mox_)^∗∗^	1205 ± 316	27 ± 18	96.1	89.1–100
	Late postpartum (PPR_Pre_)^∗∗^	4168 ± 1417	136 ± 64	96.7	85.9–99.5
	Preweaning (PW_Per_)^∗∗^	2582 ± 1205	193 ± 140	92.5	53–98.8

*T*_1_: late pregnancy treated group; *T*_2_: 48 hours postlambing treated group; PE_Mox_: posttreatment efficacy induced by moxidectin; PPER_Pre_: PPER prevention induced by moxidectin; PW_Per_: preweaning moxidectin persistency; FEC: fecal egg count; SEM: standard error of mean; FECR: fecal egg count reduction; 95% CI: 95% confidence intervals. ^**∗**^Three C ewes with high FEC and associated clinical signs were excluded after intermediate postpartum period due to Mox treatment by ethical considerations and their data were not included in the analysis. ^**∗∗**^Significant effect of treatments (one-way ANOVA and Tukey tests, *p* < 0.05) in FEC by evaluated period.

**Table 2:** Treatment-induced differences in the median of ratio change of clinical (FAMACHA© and Dag score) and zootechnical (body weight and body condition) parameters.

Measured parameters	Median (min–max)		
	Untreated control group *n* = 11^∗^	Late pregnancy Mox injection *n* = 15	48 hours postlambing Mox injection *n* = 14
Change ratio			
FAMACHA^∗∗^	0.73 (0.5–1)^a^	1 (0.67–1.25)^b^	0.67 (0.67–1)^a^
Dag score^∗∗^	1.75 (0.5–2)^a^	1 (0.67–3)^b^	1.16 (0.67–2)^ab^
Body weight	1.19 (1.06–1.28)	1.16 (1.02–1.4)	1.19 (1.05–1.42)
Body condition	1.04 (0.82–1.03)	1 (0.7–1.2)	1 (0.67–1.3)

Data were calculated by dividing the median of the parameter values before treatment (dry, mating, midpregnancy, and late pregnancy) over its median after treatment (early, intermediate, late, and preweaning period). Min: minimum value; max: maximum value; FAMACHA© values (1–5); Dag score values (0–5); body condition values (1–5). ^∗^Three C ewes with high FEC and associated clinical signs were excluded after intermediate postpartum period due to Mox treatment. ^∗∗^Significant differences between treatments (one-way ANOVA, *p* > 0.05). Treatment differences (Tukey test, *p* > 0.05) are denoted by superscripts.
